# Zinc Therapy in Mild Cognitive Impairment: Cognitive Stabilization in Pharmacodynamically Responsive Patients in the ZINCAiD Trial

**DOI:** 10.3390/biom15091268

**Published:** 2025-09-01

**Authors:** Rosanna Squitti, Alberto Benussi, Silvia Fostinelli, Andrea Geviti, Jasmine Rivolta, Mariacarla Ventriglia, Alessandra Micera, Mauro Rongioletti, Roberta Ghidoni, Matteo Santilli, Alberto Granzotto, Alberto Albanese, Giuliano Binetti, Stefano L. Sensi, Barbara Borroni

**Affiliations:** 1 Molecular Markers Laboratory, IRCCS Istituto Centro San Giovanni di Dio Fatebenefratelli, 25125 Brescia, Italy; sfostinelli@fatebenefratelli.eu (S.F.); rghidoni@fatebenefratelli.eu (R.G.); gbinetti@fatebenefratelli.eu (G.B.); bborroni@fatebenefratelli.eu (B.B.); 2 Department of Laboratory Science, Research and Development Division, Ospedale Isola Tiberina Gemelli Isola, 00186 Rome, Italy; maurociroantonio.rongioletti@fbf-isola.it; 3 Department of Theoretical and Applied Sciences (DISTA), eCampus University, 22100 Como, Italy; 4 Neurology Unit, Department of Medical, Surgical and Health Sciences, University of Trieste, 34127 Trieste, Italy; benussialberto@gmail.com; 5 Neurology Clinic, Department of Medicine, Surgery and Health Sciences, Azienda Sanitaria Universitaria Giuliano Isontina, 34148 Trieste, Italy; 6 Service of Statistics, IRCCS Istituto Centro San Giovanni di Dio Fatebenefratelli, 25125 Brescia, Italy; ageviti@fatebenefratelli.eu; 7 Neurology Unit, Department of Continuity of Care and Frailty, ASST Spedali Civili Hospital, 25123 Brescia, Italy; jasmine.rivolta@gmail.com; 8 Clinical Research Center, Ospedale Isola Tiberina Gemelli Isola, 00186 Rome, Italy; mariacarla.ventriglia@fbf-isola.it; 9 Research and Development Laboratory for Biochemical, Molecular and Cellular Applications in Ophthalmological Science, IRCCS–Fondazione Bietti, 00184 Rome, Italy; alessandra.micera@fondazionebietti.it; 10 Department of Neuroscience, Imaging and Clinical Sciences, “G. d’Annunzio” University of Chieti-Pescara, 66100 Chieti, Italy; matteblanco@hotmail.com (M.S.); alberto.granzotto@unich.it (A.G.); 11 Institute for Advanced Biomedical Technologies (ITAB), “G. d’Annunzio” University of Chieti-Pescara, 66100 Chieti, Italy; 12 Centre for Advanced Studies and Technology (CAST), “G. d’Annunzio” University of Chieti-Pescara, 66100 Chieti, Italy; 13 Neurology Institute, SS Annunziata University Hospital, 66100 Chieti, Italy; 14 Department of Neurology, IRCCS Humanitas Research Hospital, 20089 Rozzano, Italy; alberto.albanese@humanitas.it; 15 Department of Neuroscience, Catholic University, 20123 Milan, Italy; 16 Department of Clinical and Experimental Sciences, University of Brescia, 25121 Brescia, Italy

**Keywords:** clinical trial, zinc therapy, copper, mild cognitive impairment, Alzheimer’s disease

## Abstract

Dysregulation contributes to Alzheimer’s disease (AD) pathophysiology. Zinc therapy promotes enterocyte copper sequestration, potentially reducing systemic copper. Individual biological responses may vary. Methods: ZINCAiD was a 24-week, randomized, double-blind, placebo-controlled phase II trial assessing zinc therapy in individuals with mild cognitive impairment (MCI) due to AD (EudraCT No.: 2019-000604-15; registered on 26 March 2020). Participants were randomized 2:1 to receive elemental zinc (135 mg/day for 12 weeks, then 65 mg/day) or placebo. Ceruloplasmin was measured at predefined intervals for safety monitoring, blinded to the investigators. Post hoc, “Zinc Responders” were defined by ≥20% reduction in ceruloplasmin at week 12. The primary cognitive endpoint was the Cognitive Composite 2 scale (CC2); secondary endpoints included MMSE and CDR-Sob. Findings: Of the 48 participants randomized, 9 discontinued, primarily due to unrelated clinical deterioration; 39 had complete ceruloplasmin data. Two serious adverse events occurred in the Placebo group. Mild gastrointestinal symptoms occurred in eight participants, with only four leading to dropout. In the primary zinc vs. placebo analysis, no significant differences emerged in cognitive outcomes. A post hoc exploratory analysis stratified participants by pharmacodynamic response: 12 individuals with MCI due to AD (31%) met the criteria for “Zinc Responder,” defined by ≥20% reduction in serum ceruloplasmin at week 12. Only Zinc Responders maintained cognitive stability over 24 weeks, whereas the combined group of Zinc Non-Responders and placebo-treated participants showed a significant decline. For the composite cognitive score (CC2), the interaction between visit and response group was significant (*p* = 0.030), with deterioration observed only in the Non-Responder + Placebo group (Δ = –2.72, *p* < 0.0001 vs. –0.71, *p* = 0.35 in Responders). Similar patterns were observed for CDR-Sob (interaction *p* = 0.017) and MMSE (trend *p* = 0.09). Interpretation: Zinc therapy stabilized cognition in a pharmacodynamically defined MCI subgroup. These exploratory findings suggest serum ceruloplasmin as a feasible biomarker of target engagement. Larger trials are needed for confirmation.

## 1. Introduction

Alzheimer’s disease (AD) is the leading cause of dementia, projected to affect over 150 million individuals worldwide by 2050. The development of anti-amyloid monoclonal antibodies—such as lecanemab and donanemab [1]—has marked a shift in the landscape of disease-modifying therapies. However, these agents have demonstrated only partial clinical efficacy and are associated with potentially serious adverse events, including amyloid-related imaging abnormalities (ARIA) [1]. Moreover, the extent of amyloid burden does not fully account for cognitive decline, highlighting the need to explore alternative pathological mechanisms in AD. Among these, copper imbalance has long been recognized as a potential contributor to neurodegeneration. Under physiological conditions, copper homeostasis is tightly regulated to avoid accumulation of labile, redox-active forms of copper capable of generating oxidative stress and promoting β-amyloid aggregation [2,3]. Meta-analyses have described a peripheral–central copper shift in AD, with systemic copper excess and cerebral copper deficiency [4]. In addition, *ATP7B* variants—causative in Wilson’s disease (WD)—have been linked to disrupted copper metabolism and increased AD risk [4,5].

Recently, *ATP7B* was also implicated in cuproptosis, a newly characterized copper-dependent form of cell death involving mitochondrial protein aggregation and iron-sulfur cluster disruption [6]. These converging lines of evidence support the hypothesis that impaired copper handling may be directly neurotoxic in a subset of AD patients, independent of—or upstream to—amyloid pathology.

Zinc therapy, a widely used treatment for WD, reduces systemic copper availability by inducing enterocyte metallothionein and blocking copper absorption. Its efficacy and safety are well established in copper-related disorders [7,8]. Nonetheless, no double-blind trials have investigated zinc in biologically defined AD populations—likely due to limited commercial incentives. A 2012 proof-of-concept study by George G. Brewer demonstrated cognitive stabilization with zinc in AD patients, despite the absence of biomarker-based selection and inclusion of individuals at more advanced disease stages [9]. The ZINCAiD trial was conceived to address this gap. Although recruitment challenges limited its scale, the study employed a methodologically rigorous design to assess whether zinc therapy could stabilize cognition in individuals with AD in the mild cognitive impairment (MCI) phase, using post hoc pharmacodynamic response as a surrogate of biological engagement.

## 2. Methods

### 2.1. Study Design and Oversight

ZINCAiD was a 24-week, multicenter, randomized, double-blind, placebo-controlled, parallel-group phase II trial aimed at evaluating the efficacy and safety of zinc therapy in MCI due to AD. Patients with MCI due to AD were recruited between June 2020 and May 2023 at the University Hospital of Brescia (Italy) and the Memory Clinic of the IRCCS Centro San Giovanni di Dio Fatebenefratelli in Brescia (Italy). Eligible participants were randomly assigned to one of the trial’s arms, consisting of a treatment group receiving zinc therapy and a placebo group taking identical placebo tablets in a 2:1 ratio.

### 2.2. Ethics Approval

The study protocol (PTC-19-602325; EudraCT No.: 2019-000604-15; registered on 26 March 2020) was approved by the local Regional Ethics Committees (approval codes: 25/2020, CE150186; 95/2020; and 02/2021), in accordance with Italian regulatory requirements, Agenzia Italiana del Farmaco (AIFA) guidelines, and the principles of the Declaration of Helsinki. Written informed consent was obtained from all participants or their legal representatives. The trial was an investigator-initiated and academically sponsored study, supported by the Alzheimer’s Association (PTC-19-602325).

### 2.3. Participants and Selection Criteria

Eligible participants were aged 60 years or older, met internationally accepted criteria for MCI due to AD [10,11], and had a Clinical Dementia Rating (CDR) [12] global score of 0.5. Additional inclusion criteria included the following: if women, to be in menopause since at least 2 years; stable presence of an informant family member; capacity of full compliance with the protocol requirements (i.e., assumption of the medicine per os, etc.); brain MRI performed within 12 months preceding or at the screening visit; evidence of cerebral amyloidosis (β-amyloid positivity) by scanning with Florbetapir (18F)-PET or positive for cerebrospinal fluid biomarkers of AD; nonceruloplasmin copper serum concentration > 1.6 μmol/L [8,13].

Exclusion criteria included diagnosis of concomitant severe or unstable diseases and disabilities that may interfere with copper metabolism, and with primary and secondary outcome evaluation, or may bias the assessment of the clinical or mental status of the subject or put the subject at special risk; concomitant severe or unstable cardiovascular diseases; concomitant primary neurodegenerative disorder besides AD, or neurological or psychiatric disorders of any etiology; clinically significant anemia; known hypersensitivity to zinc sulphate tablets including any of the components of the formulation.

### 2.4. Randomization and Masking

Participants were randomly assigned in a 2:1 ratio to receive zinc or placebo using a computer-generated permuted block randomization sequence, stratified by site. The random allocation sequence was prepared by an independent statistician who was not involved in participant enrolment or outcome assessment. Allocation was concealed using centralized communication with site pharmacies, and all investigators, participants, and outcome assessors were blinded to treatment assignment throughout the study.

Unlike prior open-label studies in copper-related disorders, where dosing was titrated to achieve pharmacodynamic targets [7,8], ZINCAiD employed a fixed-dose, double-blind design. Ceruloplasmin (Cp) was measured at prespecified time points solely for safety monitoring. Post hoc, after unblinding, participants with a ≥20% reduction in serum Cp at week 12 were classified as Zinc Responders to explore biological engagement with therapy.

### 2.5. Procedures

Participants assigned to the zinc treatment arm received oral elemental zinc in tablet form (200 mg zinc sulfate tablets). During the first week, participants received 67.5 mg/day of elemental zinc (administered as one and a half 200 mg zinc sulfate tablets daily). From week 2 to week 12, the dose was increased to 135 mg/day (three 200 mg tablets daily, divided into three doses). Beginning at week 13 and continuing through week 24, the dose was reduced to 67.5 mg/day (one and a half 200 mg tablets daily), administered in two doses (45 mg in the morning and 22.5 mg in the evening).

Participants in the placebo group followed an identical schedule with matching placebo capsules.

Cognitive assessments were administered at baseline and 24-week visit using a standardized neuropsychological battery. Clinical safety and tolerability were evaluated at each scheduled visit—baseline visit, 3-week visit, 6-week visit, 12-week visit, and 24-week visit—through physical examination, vital signs, hematological and biochemical monitoring (including serum Cp), and adverse event reporting. Ceruloplasmin concentrations below 10 mg/dL were prespecified as a safety threshold, but no such cases were observed. In line with the double-blind design, zinc dosage was not modified based on individual laboratory results.

### 2.6. Assessments and Outcome Measures

Cognitive functions were assessed using a standardized neuropsychological battery administered at baseline and 6 months. The battery includes, as a primary cognitive outcome, the Cognitive Composite 2 scale (CC2) [14] (composed of the ADAS-3 and the cognitive portion of Clinical Dementia Rating–Sum of Boxes) [12]. The secondary cognitive efficacy outcome measures were the Clinical Dementia Rating scale sum of Boxes (CDR-SoB) [12]; the Mini-Mental State Examination (MMSE) [15]. For safety, vital signs, Cp, and blood cell count (with formula) to monitor possible anemia were administered at each visit. Physical and neurological exams and an electrocardiogram were administered at BL. Safety was also assessed by monitoring and recording adverse events (AEs) and serious adverse events (SAEs).

Serum Cp concentration was measured using a validated immunoturbidimetric assay (Futura System S.r.l., Rome, Italy) on the Pentra 400 automated clinical chemistry analyzer (Horiba ABX, Montpellier, France). Calibration and quality control procedures used pooled healthy reference sera (IMCON and INCOMLOW; Futura System S.r.l.), following manufacturer guidelines.

### 2.7. Statistical Methods

#### 2.7.1. Sample Size Calculation

To determine the necessary sample size, we based our estimation on CC2, a composite outcome combining both cognitive (ADAS-Cog) and functional (CDR-SOB) measures [14]. This composite scale captures domains most vulnerable to early neurodegeneration and is, therefore, a sensitive marker for treatment effects in patients with MCI [14]. We referred to effect sizes reported by George G Brewer [9], who observed a Cohen’s d of 0.69 for ADAS-Cog and 0.72 for CDR-SOB in a 6-month randomized trial comparing zinc therapy to placebo in AD patients. The ZINCAiD trial differed from Brewer’s study in two clinically meaningful ways that could plausibly enhance treatment responsiveness: (i) It had stricter inclusion criteria. Brewer’s sample included general AD patients without biomarker selection. In contrast, ZINCAiD recruited individuals with both elevated nonceruloplasmin copper (>1.6 μmol/L) and β-amyloid positivity—biomarkers identifying a subgroup more likely to benefit from zinc modulation. (ii) It targeted an earlier disease stage. While Brewer enrolled patients with established AD, ZINCAiD targeted the prodromal stage (MCI), where intervention is more likely to preserve function before irreversible neurodegeneration occurs. Despite these potentially favorable differences, we adopted a conservative approach using the lower of Brewer’s reported effect sizes (d = 0.69) for power estimation. Based on α = 0.05, 80% power, and a 2:1 treatment-to-placebo allocation, the required sample size was calculated at 60 participants (40 in the treatment group, 20 in the placebo group) using G*Power (version 3.1). This ensured sufficient power to detect a medium-to-large effect, while recognizing that, given the specific characteristics of our sample, a somewhat enhanced treatment effect was considered plausible.

#### 2.7.2. Statistical Analysis

Normality of continuous variables was assessed through both statistical tests and visual inspection of distribution plots. Descriptive statistics were calculated to summarize baseline characteristics. Between-group comparisons of continuous variables were conducted using independent samples *t*-tests or their non-parametric alternatives, as appropriate. Categorical variables were compared using chi-squared tests or Fisher’s Exact test, depending on expected cell counts. Longitudinal changes from baseline visit to 24-week visit were analyzed using mixed-effects models for repeated measures, including group-by-visit interaction terms to evaluate differential treatment effects over time. All models were adjusted for baseline values. Model diagnostics included assessments for outliers, residual normality, and heteroskedasticity. Under the missing-at-random (MAR) assumption, mixed-effects models implicitly handle missing outcome data, accommodating incomplete follow-up without imputation. Two analyses were performed. The primary analysis compared the original randomized groups: zinc therapy vs. placebo. The other was a post hoc exploratory analysis in which participants were grouped according to their biological response: (A) participants receiving zinc who exhibited a ≥20% reduction in ceruloplasmin levels between baseline and 12-week visit, high-dose phase; (B) all other participants, including non-responders and those in the placebo group. The decision to stratify participants based on pharmacodynamic response was grounded in previous evidence showing that a ≥20% reduction in serum ceruloplasmin reflects a biologically meaningful threshold for zinc-induced copper depletion [16,17,18]. This classification follows the rationale outlined in Section 5.3 of the EMA guidelines [19], which supports the identification of biologically plausible subgroups in exploratory analysis when underpinned by a sound mechanistic rationale and external evidence. Statistical findings reinforced this approach: A one-way ANOVA on mean percent change in Cp showed a strong group effect [F(2,36) = 50.42, *p* < 0.0001)], with post hoc Tukey HSD tests revealing no significant difference between Non-Responders and Placebo (mean difference = 0.25%, *p* = 0.998), but large, highly significant differences between Responders and Placebo (mean difference = 35.74%, *p* < 0.0001) and Responders and Non-Responders (mean difference = 35.49%, *p* < 0.0001). These results indicate that Non-Responders and Placebo participants share similar pharmacodynamic profiles, and justify their aggregation into a unified comparator group. This classification strategy is consistent with the exploratory nature of the study, aimed at estimating treatment effects specifically in biologically engaged participants [19]. Where appropriate, *p*-values were adjusted for multiple comparisons using the Bonferroni method. All statistical analyses were conducted in R (version 4.3.2). A two-sided *p*-value < 0.05 was considered statistically significant.

### 2.8. Role of the Funding Source

The study was funded by the Alzheimer’s Association Part the Cloud: Translational Research Funding for Alzheimer’s Disease (PTC) PTC-19-602325, EudraCT 2019-000604-15, through a competitive peer-reviewed grant. The sponsor had no role in the design, conduct, analysis, interpretation, or reporting of the study.

## 3. Results

### 3.1. Participants

**Figure 1:**
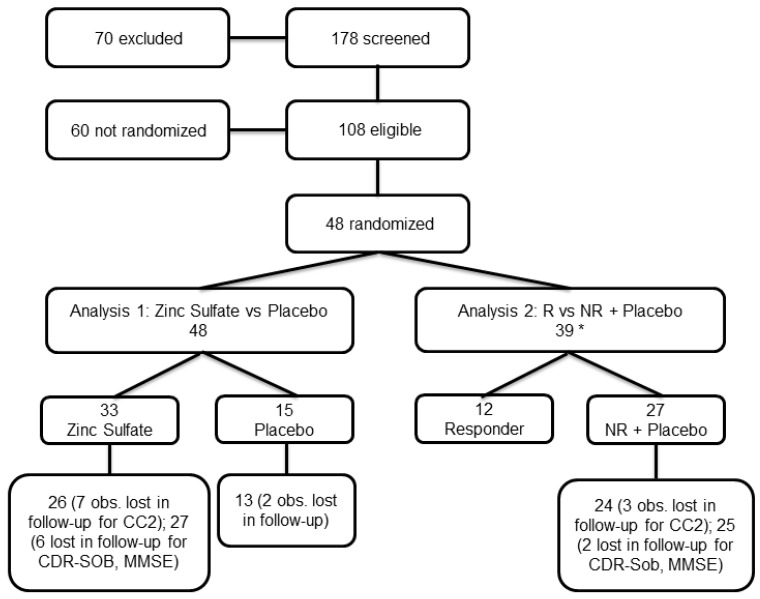
CONSORT flow diagram of the ZINCAiD trial. The figure summarizes participant flow from initial screening through randomization, treatment allocation, follow-up, and final analysis. Notably, a high screen failure rate was observed due to stringent inclusion criteria, particularly the requirement of confirmed amyloid positivity. Participant retention during the 24-week double-blind phase was acceptable. This figure reflects the final sample used for primary and secondary outcome analyses. * 39 were included in Analysis 2 since 9 subjects had Cp missing data at baseline visit.

Patients were recruited between June 2020 and May 2022. Screening, randomization, and follow-up procedures are summarized in Figure 1.

### 3.2. Baseline Characteristics and Results for Efficacy Outcomes

**Figure 2:**
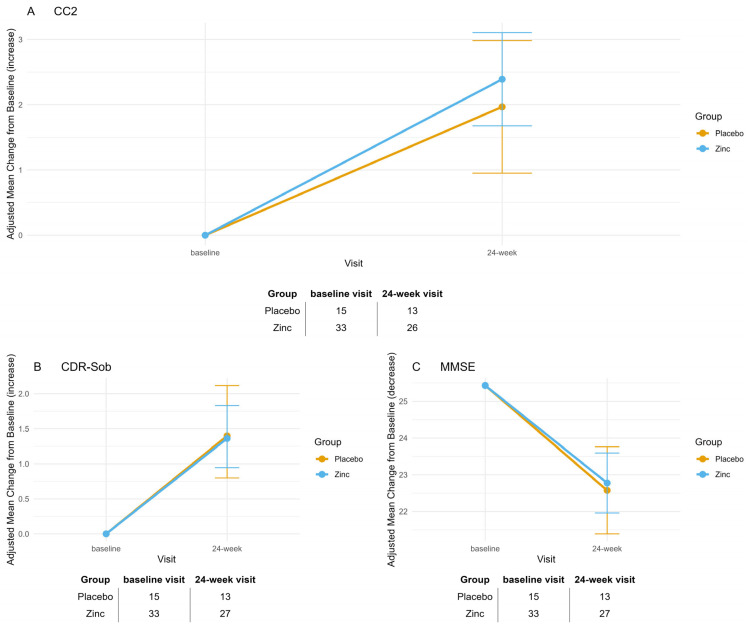
Adjusted mean change from baseline to week 24 on cognitive outcome measures (primary analysis). (**A**) Composite Cognitive Score (CC2), (**B**) Clinical Dementia Rating–Sum of Boxes (CDR-SOB), and (**C**) Mini-Mental State Examination (MMSE). Values represent adjusted mean change from baseline to 24-week visit, derived from mixed-effects models for repeated measures, with vertical bars indicating 95% confidence intervals. Data are shown for the two randomized groups: placebo (orange) and zinc sulfate (blue). Sample sizes at the baseline visit and 24-week visit are reported below each panel. A trend toward reduced cognitive decline was observed in the zinc sulfate group across all measures, with the most notable difference in CC2. Both groups declined significantly over time on all outcomes (Zinc: CC2 *p* < 0.001, Placebo: *p* = 0.019; CDR-Sob & MMSE: *p* < 0.001 for both). However, between-group differences were not significant (CC2: *p* = 1; CDR-Sob: *p* = 1; MMSE: *p* = 1).

Baseline characteristics for Analysis 1, comparing randomized groups (zinc treatment vs. placebo), are summarized in Table S1. Only MMSE scores and Cp levels differed significantly at baseline between the treatment and placebo groups. In our study, CC2 ranged from 7.6 to 33, CDR-Sob from 0.5 to 14, MMSE from 9 to 30, and Cp from 20.1 to 42.1 mg/dL. Mean changes from baseline to 24-week visit for the primary and secondary efficacy outcomes—CC2, CDR-Sob, and MMSE—are reported in Table S2 and illustrated in Figure 2. Both groups showed significant deterioration over time across all outcomes (CC2: *p* < 0.001 for Zinc, *p* = 0.019 for Placebo; CDR-Sob: *p* < 0.001 for both groups; MMSE: *p* < 0.001 for Zinc, *p* = 0.002 for Placebo). The mean differences between treatment and placebo groups were not statistically significant for any outcome (A: CC2, *p* = 1; B: CDR-Sob, *p* = 1; C: MMSE, *p* = 1). Bonferroni adjustment was applied to all *p*-values to account for the three assessed outcomes.

**Figure 3:**
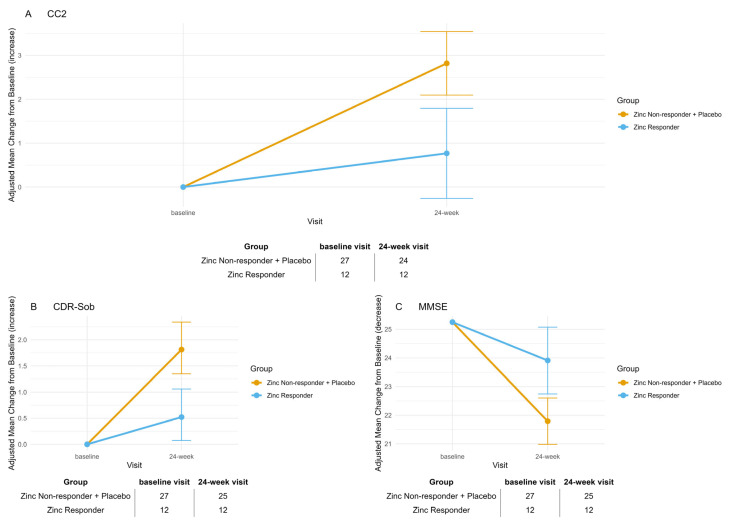
Adjusted mean change from baseline to week 24 in Zinc Responders versus Placebo/Non-Responders (post hoc pharmacodynamic subgroup analysis). (**A**) Composite Cognitive Score (CC2), (**B**) Clinical Dementia Rating–Sum of Boxes (CDR-SOB), and (**C**) Mini-Mental State Examination (MMSE). Participants in the Zinc Responder group were defined by a ≥20% decrease in ceruloplasmin from baseline to 12-week visit, reflecting effective pharmacodynamic engagement. Data show adjusted mean changes with 95% confidence intervals, estimated from mixed-effects models for repeated measures. Sample sizes at the baseline visit and 24-week visit are reported below each panel. Zinc Responders exhibited a stabilization or improvement in all cognitive endpoints, in contrast to the decline observed in the combined group of zinc Non-Responders and Placebo.

The reduction in sample size (from 60 randomized subjects to 48 analyzed) inevitably lowered statistical power, complicating the interpretation of non-significant results in the primary comparison. To contextualize this limitation, we conducted a power calculation using G*Power based on the expected effect size (Cohen’s d = 0.69). With α = 0.05 and n = 48 (Zinc = 33, Placebo = 15) for an independent-samples *t*-test, the achieved power was approximately 70%. Baseline characteristics for Analysis 2 (Zinc Responders vs. Non-Responders plus Placebo) are presented in Table 1. A total of 39 participants had available Cp measurements at both baseline and 12-week visit. Among those receiving zinc, 12 participants had a biological response, defined as a ≥20% reduction in Cp levels, and were classified as Responders. The remaining treated participants were classified as Non-Responders and grouped with placebo participants. Mean changes from baseline to week 24 for the same efficacy outcomes are shown in Table 2 and Figure 3. For all outcomes, the Responder group remained stable over time, showing no significant within-group change (CC2: *p* = 0.882; CDR-Sob: *p* = 0.298; MMSE: *p* = 0.327), whereas the combined Non-Responder + Placebo group exhibited highly significant deterioration across all endpoints (CC2: *p* < 0.001; CDR-Sob: *p* < 0.001; MMSE: *p* < 0.001). Between-group comparisons confirmed statistically significant differences in change from baseline at week 24, with Responders showing less decline compared to Non-Responders + Placebo (CC2: Δ = –2.05, SE = 0.63, *p* = 0.006; CDR-Sob: Δ = –1.29, SE = 0.35, *p* = 0.002; MMSE: Δ = 2.12, SE = 0.71, *p* = 0.012). Bonferroni adjustment was applied to all *p*-values to account for the three assessed outcomes.

**Figure 4:**
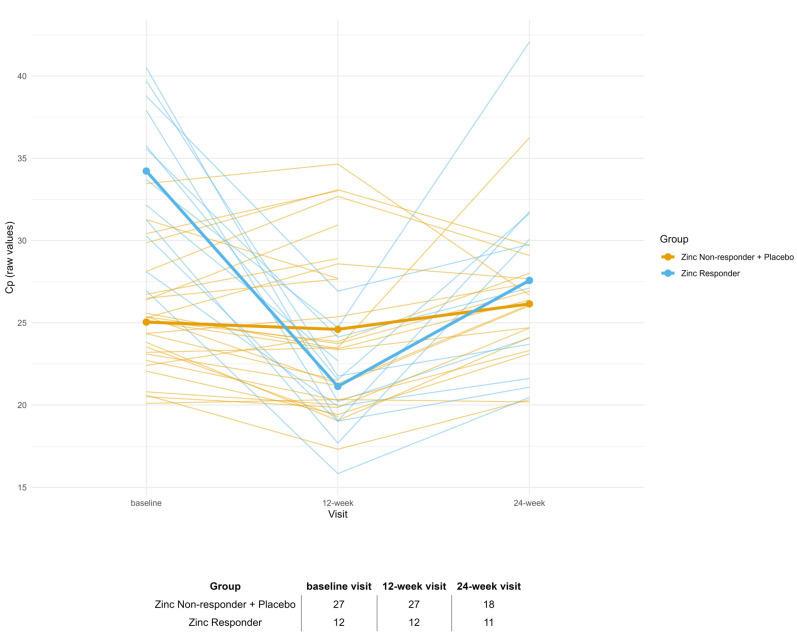
Individual trajectories of ceruloplasmin (Cp) across time (stratified by Cp group). The expected drop in Cp at 12-week visit (maximum dosage) in the Zinc Responder group supports the biological validity of the responder definition.

The decline in Cp levels from baseline to week 12-week visit in Zinc Responders, as well as the stability of Cp in Non-Responders and the Placebo group, is shown in Figure 4.

### 3.3. Safety and Study Discontinuation

Of the participants enrolled, nine discontinued the study before study completion. The most common reason was patient withdrawal due to clinical deterioration judged unrelated to the study drug. Two serious adverse events (one ischemic stroke and one acute myocardial infarction), both occurring in the placebo group, also led to treatment discontinuation. No serious adverse events occurred in the zinc group. Other reasons for discontinuation included logistical limitations (e.g., inability to attend visits or undergo venous sampling) and personal choice. Mild adverse events were reported in eight participants, most commonly appetite loss, nausea, vomiting, epigastric burning, and abdominal discomfort. These events were transient, managed symptomatically, and did not require discontinuation of treatment. Notably, only four participants who experienced mild adverse events subsequently chose to withdraw from the study, indicating that these effects were not the primary reason for dropout in most cases. Moreover, regarding Analysis 2, nine patients had Cp missing data at baseline because the protocol did not require the assessment of Cp at baseline.

## 4. Discussion

The randomized, placebo-controlled clinical trial ZINCAiD provides preliminary, biologically anchored evidence that zinc therapy may stabilize cognitive function in a pharmacodynamically defined subset of MCI patients, specifically, those having a ≥20% reduction in serum ceruloplasmin [16,17,18,20]. Despite the limited sample size and lack of efficacy in the primary zinc vs. placebo comparison, a consistent pattern emerged across three clinical scales (MMSE, CC2, and CDR-SOB): Only patients on zinc who reached a pharmacodynamically relevant copper-deficient state maintained cognitive stability, while all others declined significantly. The rationale for targeting zinc therapy to a copper-related metabolic phenotype derives from a long-standing hypothesis that implicates elevated non-ceruloplasmin copper (also called “free copper” or exchangeable copper [13]) as a contributor to neurotoxicity in AD [9,21]. Although only exchangeable copper was measured in this study, we acknowledge the terminological distinctions commonly used in the literature. “Non-ceruloplasmin copper” (non-Cp Cu) is an indirect estimate derived by subtracting ceruloplasmin-bound copper—calculated from serum ceruloplasmin concentrations using the Walshe formula—from total copper. “Exchangeable copper,” by contrast, is measured directly through ultrafiltration and reflects copper loosely bound to low-affinity carriers such as albumin and amino acids. The term “free copper” is often used synonymously with non-Cp Cu, although truly unbound ionic copper likely exists only at attomolar levels. Despite their differing methodologies, our previous work has shown that non-Cp Cu and exchangeable copper provide functionally equivalent information in characterizing copper dysregulation in AD [13].

Zinc acts by inducing metallothioneins in enterocytes, thereby blocking intestinal copper absorption and gradually depleting systemic excess copper levels [22,23,24].

The cognitive stabilization observed in those patients who reached a pharmacodynamic state of chemical copper deficiency supports the hypothesis that copper dysregulation may be a tractable contributor to disease progression in this subgroup—an insight deeply rooted in the clinical experience of Brewer and Hoogenraad, who treated hundreds of patients with copper metabolism disorders using zinc-based therapy [9,25]. Indeed, the associated reduction in serum ceruloplasmin levels reflects a pharmacodynamically meaningful decline in bioavailable copper—sufficient to affect biological pathways implicated in disease progression [17]. This effect aligns with previous studies showing rapid ceruloplasmin decrease in response to high-zinc diets or anti-copper therapy [16,17]. Ceruloplasmin is widely recognized as a reliable pharmacodynamic marker of copper depletion in both Wilsonian and non-Wilsonian contexts. In such conditions, a ≥20% reduction in serum ceruloplasmin is used as a surrogate for systemic copper depletion and correlates with biological target engagement [16,17,18]. We therefore classified as Zinc Responders those individuals showing a ≥20% reduction after 12 weeks of full-dose zinc. This threshold is biologically justified, as prior studies confirmed that such a reduction reflects effective hepatic copper depletion via metallothionein induction [7,16,17]. Although zinc was administered at a fixed dose due to the double-blind design, a substantial proportion of participants still reached the pharmacodynamic threshold, indicating that 135 mg/day elemental zinc was sufficient to induce copper depletion in responsive individuals. However, some did not show this response, underscoring interindividual variability and suggesting that only a subset may reach the therapeutic window under fixed dosing. This supports the value of dose titration in future trials. Serum ceruloplasmin may also serve as a low-cost, minimally invasive biomarker to personalize treatment and facilitate broader implementation in phase II studies.

Our data suggest that pharmacodynamic copper depletion, as indicated by ≥20% reduction in ceruloplasmin, is a necessary condition for cognitive stabilization under zinc therapy. However, target engagement alone may not be sufficient in all cases to guarantee clinical benefit, as additional biological or clinical factors may influence the therapeutic response. These findings raise important questions regarding how best to monitor and interpret biological response in the context of a blinded, fixed-dose intervention. A key methodological limitation is the incompatibility between pharmacodynamic dose titration and double-blind design in our exploratory, non-commercial study. In open-label studies of anti-copper therapy, including those by Brewer and Hoogenraad [7,8], zinc doses were adjusted using real-time ceruloplasmin monitoring to achieve individualized copper depletion. By contrast, ZINCAiD used a fixed-dose, double-blind design to preserve internal validity, which precluded dose adjustment during the trial. Consequently, target engagement was assessed only retrospectively, identifying a biologically defined subgroup post hoc, as permitted by EMA guidelines Section 5.3 [19]. To avoid unblinding through pharmacodynamic monitoring, a Cp-guided titration strategy would require an open-label or adaptive trial design—both more complex and resource-intensive than the current randomized controlled framework.

While our study was not powered to prospectively validate this biological stratification, the consistency between pharmacodynamic response and clinical trajectory provides a compelling rationale for future targeted trials.

The increase in ceruloplasmin levels observed at the 12-week visit among Zinc Responders is consistent with the protocol-defined halving of zinc dosage after Week 12, from 135 mg/day to 65 mg/day. This pharmacodynamic rebound likely reflects the dose-dependent induction of intestinal metallothionein, which is critical for zinc-mediated copper sequestration [16,17]. Lower zinc exposure may reduce metallothionein expression, leading to increased copper absorption and a subsequent rise in ceruloplasmin synthesis. These findings highlight the need to maintain adequate zinc dosing to sustain copper control, especially in individuals with biologically proven responsiveness.

The correlation between the biochemical engagement and cognitive stabilization in Zinc Responders reinforces the hypothesis that modulating copper metabolism may influence trajectories in biologically selected MCI patients. This association was further supported by converging results across two validated clinical endpoints—CDR-SOB and CC2—allowing comparison between a widely adopted, semi-structured staging tool and a neuropsychological composite designed for sensitivity to early cognitive changes [14,26]. CDR-SOB is widely used in MCI trials for disease staging [1,27], but its semi-structured format may limit sensitivity to subtle pharmacological effects [26]. By contrast, our primary outcome, CC2 [14], combines standardized neuropsychological tests into a quantitative index tailored to detect small yet clinically meaningful changes. In our study, CC2 showed significant cognitive stability in Zinc Responders over 24 weeks, with effect sizes exceeding those typically seen with CDR-SOB. For instance, the Lecanemab phase 3 trial reported a ~0.45 CDR-SOB difference at 18 months [1], while Koch et al. observed a ~0.7 reduction after 6 months of stimulation in Aβ-positive MCI [27]. These effects were modest and based on global measures [1,9,27]. ZINCAiD prioritized a cognitive composite increasingly adopted in early-phase AD trials [14], and the concordant improvements in CC2 and CDR-SOB among responders reinforce the observed treatment effect.

The emerging concept of cuproptosis—a copper-driven, regulated cell death pathway—provides a mechanistic link between systemic copper dysregulation and neurodegeneration. Triggered by excess copper binding to lipoylated tricarboxylic acid cycle enzymes, it induces proteotoxic stress, mitochondrial dysfunction, and cell death [6]. Initially described in cancer, cuproptosis is now being explored in neurodegenerative disorders, including AD, where mitochondrial vulnerability is a hallmark. In this light, the cognitive stabilization seen in Zinc Responders may stem from zinc-induced metallothioneins sequestering copper and limiting mitochondrial accumulation. By reducing cuproptosis susceptibility, zinc may confer neuroprotection in copper-related phenotypes. Though direct evidence in the human brain is lacking, the association between biochemical engagement (≥20% Cp reduction) and clinical benefit in our study supports its relevance. Future trials with mechanistic biomarkers (e.g., mitochondrial stress, lipoylated aggregates) may clarify cuproptosis’ role in MCI and treatment response.

Our findings are consistent with previous trials suggesting zinc may attenuate cognitive decline in AD and MCI. Studies have reported cognitive stabilization—especially in older adults or individuals with altered copper metabolism [9,28]. In a 6-month study of patients over 70 with probable AD, Brewer showed that zinc acetate slowed decline on ADAS-Cog and CDR-SOB, with similar trends in MMSE [9]. ZINCAiD built on this by applying biomarker-based stratification and enrolling participants at the prodromal stage, enabling a more targeted assessment of zinc’s pharmacodynamic and cognitive effects.

Zinc’s neuroprotective effects stem not only from correcting copper imbalance but also from direct actions on synaptic and cellular processes, including N-methyl-D-aspartate (NMDA) receptor modulation and oxidative stress reduction [29,30]. In traumatic brain injury, zinc therapy has been associated with reduced mortality and improved Glasgow Coma Scale scores in a 1-month trial, while a 2018 phase II randomized controlled trial reported superior clinical and inflammatory outcomes in zinc-treated patients compared to placebo. These findings underpin the 2022 Canadian Network for Mood and Anxiety Treatments (CANMAT) guideline, which recommends zinc—with Level 2 evidence—for cognitive and psychiatric support [31].

The ZINCAiD protocol was designed under the scientific mentorship of George Brewer, a pioneer in copper biology and zinc therapy [7,9,16,17,21,24], and builds on decades of laboratory and clinical data suggesting a role for metal homeostasis in AD [9,21]. The philosophical and mechanistic foundation of this work is also closely aligned with the vision of T.U. Hoogenraad, whose contributions to the field have been recognized [32]. This study, in many ways, represents a clinical continuation of that therapeutic perspective [32]. In line with the hypothesis first proposed by T.U. Hoogenraad, some forms of AD may represent a senile variant of copper toxicosis, akin to a late-onset or heterozygous form of WD [25], and reflect overlapping mechanisms with WD [5]. This conceptual shift invites reconsideration of AD not only as a neurodegenerative disorder but also as a condition in which trace metal imbalance contributes to disease progression in a susceptible subset of individuals. From this perspective, the clinical stabilization observed in zinc “Responders” in the ZINCAiD trial—despite the limited sample size—may reflect a therapeutic benefit similar to that observed in WD under long-term zinc therapy. While such a proposition remains speculative, it raises the possibility that treatments validated in copper toxicosis could be repurposed for use in selected AD, guided by pharmacodynamic markers such as ceruloplasmin. This approach, if confirmed, may complement traditional trial frameworks with a precision medicine model.

While our study focused on zinc sulfate due to its favorable safety profile and clinical use in WD, alternative copper-lowering agents such as tetrathiomolybdate (TM) also warrant consideration. TM exerts dual action as a potent copper chelator and anti-angiogenic compound, and it has shown promise in oncology and neurodegeneration [17,33]. Comparative studies between zinc and TM could help elucidate differences in mechanisms of action, biomarker modulation, and cognitive outcomes in AD. Future head-to-head or combinatorial trials may provide valuable insights for optimizing patient-specific therapeutic strategies. In addition, the pharmacodynamic responsiveness observed in the ZINCAiD study may define a therapeutically relevant biological patient subgroup. Building on this pharmacodynamic distinction, emerging therapeutic strategies are now being explored to restore metal homeostasis with increased selectivity and mechanistic precision. Emerging strategies to correct brain copper and zinc dyshomeostasis in AD include peptide-mediated metal shuttles, nanoparticle-based delivery systems, and nutraceuticals with redox-active or metal-modulating properties. A recent study introduced a Cu(II) shuttle based on a cell-penetrating peptide that delivers bioavailable copper via Rab5- and Rab14-mediated endocytosis, reducing Cu–Aβ-induced oxidative stress [34]. Zinc-based compounds such as zeolite–zinc improved memory and hippocampal integrity in Aβ-treated animals [35], while hydroxytyrosol altered zinc and copper distribution in a transgenic AD mouse model [36]. Furthermore, a clinical study combining olive polyphenols and S-acetyl-glutathione showed stabilization or improvement in cognitive function in patients with mild AD [37]. Collectively, these findings support the development of targeted, mechanism-driven interventions acting on metal homeostasis and oxidative stress in early AD.

The primary limitation of the ZINCAiD trial is the modest sample size, which—while sufficient to detect a medium-to-large treatment effect—may limit the generalizability of the findings and the ability to explore subgroup effects. Although the a priori power analysis supported the planned sample size, the final enrolled cohort was smaller than expected. As such, the possibility of a type II error—failing to detect a true effect due to insufficient power—cannot be excluded, particularly in the primary comparison. A post hoc estimate based on the expected effect size (Cohen’s d = 0.69) and observed group sizes yielded a power of approximately 70.5%. While this is moderately acceptable, the results should be interpreted with caution, and future studies with larger samples will be essential to confirm these findings and enable more granular subgroup analyses. Additional limitations include the fixed-dose design, which did not allow for personalized zinc titration to ensure that all participants reached the targeted ceruloplasmin reduction, and the binary classification of “responders” based on a post hoc threshold—even though this threshold was pharmacodynamically justified and grounded in prior anti-copper therapy research. Furthermore, we did not incorporate longitudinal imaging or CSF biomarkers beyond the screening phase. Nevertheless, the consistency of clinical stabilization across multiple independent cognitive outcomes, coupled with a biologically coherent pharmacodynamic signal, strengthens the internal validity of the observed effects. This work suggests that biologically stratified therapy targeting copper metabolism may offer a low-cost, mechanism-driven approach to slowing cognitive decline in MCI. Future trials should aim to replicate these findings in larger, multicenter cohorts, using early ceruloplasmin reduction as both a marker of target engagement and a potential enrichment strategy for identifying treatment responders.

## 5. Conclusions

In this randomized, double-blind trial, oral zinc therapy stabilized cognition in a pharmacodynamically defined subgroup of MCI patients with systemic copper imbalance. These findings support the role of copper homeostasis as a therapeutic target in early AD and propose serum ceruloplasmin as a feasible pharmacodynamic marker. Despite the limited sample size, the alignment between biochemical and clinical outcomes warrants larger confirmatory trials within a precision medicine framework. The results also revive the hypothesis of trace metal dysregulation in AD and suggest that anti-copper strategies used in WD may be applicable in selected neurodegenerative cases.

## Figures and Tables

**Table 1:** Analysis 2: baseline characteristics.

	Total (n = 39)	R (n = 12)	P + NR (n = 27)	*p* Value *
Gender	M = 27 (69%)	M = 10 (83%)	M = 17 (63%)	0.276
Age	71 (7.3)	68.7 (10.4)	72.0 (5.4)	0.680
Onset Age	67.5 (7.3)	65.7 (10.1)	68.3 (5.8)	0.843
CC2	18.7 (3.2)	18.3 (3.4)	18.9 (3.1)	0.607
CDR-Sob	2.2 (0.9)	2.0 (0.8)	2.3 (1.0)	0.276
MMSE	25.2 (1.5)	25.2 (1.2)	25.2 (1.6)	0.875
Cp (mg/dL)	27.9 (5.7)	34.2 (4.6)	25.0 (3.4)	<0.001

P = Placebo, R = Responders, and NR = Non-Responders; * Fisher Exact test for gender, *t*-test for CC2, and Mann–Whitney U test for the other variables.

**Table 2:** Analysis 2: primary and secondary end points.

End Point	Mean Change R (n = 12)	Mean Change NR + P (n = 27)	Mean Difference in Change vs. NR + P (SE)	*p* Value ^$^
Primary efficacy endpoint Mean change from baseline to week 24 in the CC2	0.77	2.82	−2.05 (0.63)	R: 0.882 NR + P: <0.001 R vs. NR + P: 0.006
Secondary efficacy endpoints Mean change from baseline to week 24 in the CDR-Sob score	0.52	1.81	−1.29 (0.35)	R: 0.298 NR + P: <0.001 R vs. NR + P: 0.002
Secondary efficacy endpoints Mean change from baseline to week 24 in the MMSE	−1.34	−3.46	2.12 (0.71)	R: 0.327 NR + P: <0.001 R vs. NR + P: 0.012

P = Placebo, R = Responders, and NR = Non-Responders. ^$^ Bonferroni adjustment was applied to all *p*-values to account for the three tests performed.

## Data Availability

The de-identified clinical dataset supporting the findings of this study is available upon reasonable request from the corresponding author. Access will be granted for academic and non-commercial use, subject to ethical approval and a formal data-sharing agreement in compliance with institutional and regulatory policies.

## Supplementary Materials

The following supporting information can be downloaded at: https://www.mdpi.com/article/10.3390/biom15091268/s1, Table S1: Analysis 1: Baseline Characteristics. Table S2: Analysis 1: Primary and Secondary End Points (Modified Intention-to-Treat Population).
