# Supplementary material for: Zinc Therapy in Mild Cognitive Impairment: Cognitive Stabilization in Pharmacodynamically Responsive Patients in the ZINCAiD Trial
Source: Biomolecules. 2025 Sep 1;15(9):1268. doi: 10.3390/biom15091268 (PMC12466942; doi:10.3390/biom15091268)
Supplement: Supplementary file 1 [file biomolecules-15-01268-s001.zip › biomolecules-3819631-supplementary.pdf]

Supplementary Materials

Table S1. Analysis 1: Baseline Characteristics

	**Total (N=48)**	**Zinc (N=33)**	**Placebo (N=15)**	***p****
Gender	M = 32 (67%)	M = 24 (73%)	M = 8 (53%)	.322
Age	71.1 (7.1)	71.0 (7.8)	71.4 (5.5)	.624
Onset Age	67.6 (7.2)	67.5 (7.7)	67.9 (6.6)	.841
CC2	18.5 (3.8)	19.0 (3.5)	17.3 (4.1)	.173
CDR-Sob	2.3 (0.9)	2.5 (1.0)	2.1 (0.8)	.209
MMSE	25.4 (1.8)	25.1 (1.6)	26.2 (2.0)	**.041**
Cp (mg/dL)	27.9 (5.7)	29.6 (5.9)	23.9 (2.3)	**.004**

*chi-squared test for gender, t-test for CC2, Mann-Whitney U test for the other variables

Table S2. Analysis 1: Primary and Secondary End Points

**End Point**	**Mean change Zinc (N=33)**	**Mean change Placebo (N=15)**	**Mean difference in change vs. placebo (SE)**	***p $***
Primary efficacy end point<br><br>*Mean change from baseline to week 24 in the CC2*	2.39	1.97	0.42 (0.63)	Zinc: ***<.001***<br>Placebo: ***.019***<br>Zinc vs Placebo: 1
Secondary efficacy end points<br><br>*Mean change from baseline to week 24 in the CDR-Sob score*	1.37	1.40	-0.03 (0.40)	Zinc: ***<.001***<br>Placebo: ***<.001***<br>Zinc vs Placebo: 1
Mean change from baseline to week 24 in the MMSE	-2.66	-2.86	0.20 (0.73)	Zinc: ***<.001***<br>Placebo: ***.002***<br>Zinc vs Placebo: 1

$ Bonferroni adjustment was applied to all p-values to account for the three tests performed.
